# Prevalence and Predictors of Antimicrobial Resistance Among *Enterococcus* spp. From Dogs Presented at a Veterinary Teaching Hospital, South Africa

**DOI:** 10.3389/fvets.2020.589439

**Published:** 2021-01-07

**Authors:** James Wabwire Oguttu, Daniel Nenene Qekwana, Agricola Odoi

**Affiliations:** ^1^Department of Agriculture and Animal Health, College of Agriculture and Environmental Sciences, University of South Africa, Johannesburg, South Africa; ^2^Section Veterinary Public Health, Department of Paraclinical Sciences, Faculty of Veterinary Science, University of Pretoria, Pretoria, South Africa; ^3^Department of Biomedical and Diagnostic Sciences, College of Veterinary Medicine, University of Tennessee, Knoxville, Knoxville, TN, United States

**Keywords:** *Enterococcus* species, enterococci, antimicrobial resistance, canine, dogs, multi-drug resistance, pan-drug resistance, extensive-drug resistance

## Abstract

**Background:** While surveillance of antimicrobial drug resistance is ongoing in human medicine in South Africa, there is no such activity being performed in veterinary medicine. As a result, there is a need to investigate antimicrobial resistance among enterococci isolated from dogs in South Africa to improve understanding of the status of antimicrobial drug resistance given its public and veterinary public health importance. This study investigated antimicrobial resistance and factors associated with resistance profiles of enterococci isolated from dogs presented for veterinary care at a veterinary teaching hospital in South Africa.

**Methods:** In total 102 *Enterococcus* isolated between 2007 and 2011 by a bacteriology laboratory at a teaching hospital were included in this study. Antimicrobial susceptibility of the isolates was determined against a panel of 18 antimicrobials using the Kirby Bauer disc diffusion technique. Univariate analysis was used to assess simple associations between year, season, breed group, age group, sex, and specimen as covariates and extensive drug resistance (XDR) as the outcome. Variables that were significant in the univariate analysis at a generous *p*-value ≤ 0.2 were included in the multivariable logistic models to investigate predictors of XDR.

**Results:** All the *Enterococcus* isolates were resistant to at least one antimicrobial. High proportions of isolates were resistant against lincomycin (93%), kanamycin (87%), orbifloxacin (85%), and aminogycoside-lincosamide (77%). Ninety three percent (93%), 35.3, and 8.8% of the isolates exhibited multi-drug, extensive-drug and pan-drug resistance, respectively. Only year was significantly (*p* = 0.019) associated with extensive-drug resistance.

**Conclusion:** Given the zoonotic potential of *Enterococcus* spp., the high antimicrobial resistance and multi-drug resistance observed in this study are a public health concern from one health perspective. The identified resistance to various antimicrobials may be useful in guiding clinicians especially in resource scarce settings where it is not always possible to perform AST when making treatment decisions.

## Introduction

Enterococci are Gram-positive, non-spore forming facultative anaerobic bacteria that can survive a wide range of temperatures, pH, hyperosmolarity, and prolonged desiccation (1–3). *Enterococcus faecalis, Enterococcus faecium*, and *Enterococcus durans*, are the most commonly isolated *Enterococcus* species from animals. However, *E. faecalis* and *E. faecium* are the most common in dogs (4–6).

The public health significance of enterococci is based on their ability to develop resistance and horizontally transfer this resistance to other bacteria (7). This is confirmed by a number of studies that have reported enterococci as an important source of resistant genes for organisms such as *Listeria* spp. (8, 9) and *Staphylococcus aureus* (10, 11). The public health importance of enterococci is exacerbated by the fact that pets are a putative reservoir of antimicrobial-resistant bacteria for humans (12).

Available evidence suggests that enterococci tend to develop resistance to most antibiotics including: penicillin, chloramphenicol, tetracyclines, rifampin, fluoroquinolones, aminoglycosides, and vancomycin (13–15). This resistance is both intrinsic and acquired (13–17). In view of this, antibiotic resistance is a problem that complicates therapy in patients with enterococcal infection, with potential to compromise successful treatment of enterococci infection (18). Resistant enterococci have also been observed in poultry and poultry abattoir workers (19, 20). Recent reports suggest that resistance among enterococci organisms from humans is not only limited to first line antimicrobials such as the beta-lactams, but has also been observed against antimicrobials such as vancomycin that are reserved for use as the second line of defense (21, 22).

Enterococci are opportunistic pathogens that form part of the normal microbial flora in the gastrointestinal tracts of humans, animals, and birds (23–27). When found outside the gut, they are indicators of fecal contamination (3). However, over the past decade, enterococci have become the leading cause of nosocomial and community-acquired infections in human medicine (18, 28–30).

Some of the conditions caused by enterococci include endocarditis, bacteraemia, urinary tract infections, neonatal sepsis (31, 32), diarrhea, biliary tract infection, peritonitis, post-operative infection, and post-partum endomyometritis (32). Moreover, enterococci have become increasingly resistant to antimicrobial agents commonly used in veterinary hospitals (18, 19, 28–30).

There is no evidence of studies in South Africa that have investigated antimicrobial resistance profiles of *Enterococcus* species in dogs. Furthermore, while surveillance of antimicrobial resistance is ongoing in human medicine in South Africa, currently there is no such activity being performed in veterinary medicine. As a result, there is a need to investigate antimicrobial resistance among enterococci isolated from dogs in South Africa. This study investigated antimicrobial resistance and factors associated with resistance profiles of enterococci isolated from dogs presented for veterinary care at a veterinary teaching hospital in South Africa. This study is based on the hypothesis that presupposes that resistance profiles of clinical enterococci isolated from dogs admitted at a veterinary teaching hospital are influenced by several host factors. This study is intended to contribute to improved understanding of antimicrobial resistance profiles of enterococci from dogs, and information from this study will enable clinicians to make decisions regarding the most appropriate and effective antimicrobial therapies to treat enterococci infections in dogs.

## Methods

### Ethical Statement

This study was approved by the Research Ethics Committee of the University of South Africa (REC Reference Number: 2019/CAES_AREC/107). When owners of animals present their animals for treatment at the Veterinary Teaching Hospital where the data were obtained, they are requested to sign a consent form granting permission for samples and data collected for the purpose of diagnosis to also be used for research purposes. To ensure anonymity and confidentiality of patients and their owners, study findings are reported in aggregated form. As a result, no patient identifiable information is included in this manuscript.

### Study Area

This retrospective data used in this study came from a laboratory associated with veterinary teaching hospital in Gauteng province, South Africa. The province spans approximately over 18,000 km^2^ and consists of rural, urban and peri-urban areas. Based on the 2019 mid-year population estimates, the province has a human population of 15,176,115 people and it is the most populated province in South Africa (33).

South Africa has been dubbed a dog country with a population of 9.1 million dogs. Residents of Gauteng province keep dogs for various reason that include: for personal security, as companions, and to keep feral cats away from their compounds (34, 35). Oosthuizen et al. (36) observed that 28% of the dogs in selected provinces of South Africa that included Gauteng Province, were stray dogs while 72% had owners. This notwithstanding, keeping dogs is regulated by city by-laws. These by-laws for example, prescribe the number of dogs that one can keep on their premises, and requires that all dog owners ensure that at all times the dog is confined to the premises of the owner (37). With respect to access to veterinary services, this is skewed in favor of dogs in urban and peri urban areas where veterinary services are readily available. However, this is not the case for dogs in rural and/or formerly disadvantaged areas (areas where the majority of black South Africans reside). In South Africa, while antibiotics used in cattle are available over the counter without a prescription, this is not the case with antibiotics use in companion animals such as dogs. In the later, antibiotics are only available by prescription from a qualified veterinarian.

Gauteng province experiences four seasons: summer (November–March), autumn (April–May), winter (June–August), and spring (September–October). Winter is the driest season, while summer (especially December and January) is the wettest season. The province experiences annual maximum temperatures of about 22°C in the south and 25°C in the north (38).

### Isolation of *Enterococcus* spp. and Testing for Antimicrobial Susceptibility

All samples used in the study were processed by the bacteriology laboratory associated with a teaching veterinary academic hospital. The laboratory follows standardized protocols for isolation of *Enterococcus* spp. described by Quinn et al. (39). Furthermore, the laboratory conducts antimicrobial susceptibility testing using the Kirby Bauer disc diffusion technique following the guidelines described by the Clinical and Laboratory Standards Institute (CLSI) (40–46). The susceptibility profile of the *Enterococcus* isolates included in this study were determined against a panel consisting of the following 18 drugs: 30 μg-amikacin, 20/10 μg amoxicillin-clavulanic acid,100 μg carbenicillin,30 μg ceftazidime, 30 μg cephalothin,30 μg chloramphenicol, 30 μg doxycycline, 5 μg enrofloxacin,10 μg gentamicin, 30 μg imipenem, 30 μg kanamycin, 2 μg lincomycin, 100 μg lincospectin,5 μg orbifloxacin, 10 μg penicillin-G, 25 μg trimethoprim_sulfamethoxazole, 10 μg tobramycin, and 15 μg tylosin tartrate (Oxoid Ltd., Cambridge, UK).

The laboratory that provided the data also follows the CLSI protocol for isolation of enterococci organisms and uses clinical breakpoints, to determine breakpoints of each isolate (40–46). However, where there are no clinical breakpoints, it adopts epidemiological cut-off points. Furthermore, where there are no veterinary break points, the laboratory uses human breakpoints.

The laboratory reports antimicrobial susceptibility profile of the isolates as susceptible, intermediate or resistant. However, the data provided did not include isolates that were classified as intermediate.

For purpose of this study, antimicrobial resistance (AMR) was defined as resistance to at least one of the tested antimicrobials. Multidrug resistance (MDR) was defined as resistance to at least one agent in more than three antimicrobial categories, while extensive drug resistance (XDR) was defined as resistance to at least one agent in all but two or fewer antimicrobial categories. Finally, pan-drug (PDR) resistance was defined as resistance to all antimicrobial categories tested (47).

### Data Source

Laboratory records of 102 clinical isolates of *Enterococcus* spp. from dogs presented at a veterinary teaching hospital between January 2007 and December 2011 were included in this study. The inclusion criteria included the following: an isolate had to have data on AMR and other explanatory variables to be included in the study or the analysis. Fields extracted from the records included: breed, sex, age, date sample was submitted, type of specimen submitted, organ system giving rise to the sample as well as culture and antimicrobial susceptibility test results.

### Data Management, Rationale of Data Analysis Approach and Data Analysis

#### Data Management

To prepare the data for analysis, dog breeds were categorized based on the American Kennel Club classification into breed groups (48). The age of dog was analyzed both as a numeric and categorical variable. As a categorical variable, age was categorized as described by Chen et al. (49), and was consequently recoded into five categories (<2, 2–4, >4–7, >7–10, and >10 years old) (Table 1). Since several types of specimen were submitted to the laboratory for isolation of enterococci, this variable was re-coded into a five-category variable.

**Table 1:** Distribution of *Enterococcus* and multidrug resistant enterococcal isolates among dog specimens tested at a teaching veterinary hospital in South Africa, 2007-2011.

		**Enterococcus isolates**			**MDR**	
**Variable**	**Category**	**Number**	**Percentage**	**x/n**	**Percentage**	*p*-value^*^
Year (*n* = 102)						0.8578
	2007	11	10.8	10/11	90.9	
	2008	17	16.7	16/17	94.1	
	2009	49	48.0	45/49	91.8	
	2010	14	13.7	14/14	100.0	
	2011	11	10.8	10/11	90.9	
Season (*n* = 102)						0.3012
	Summer	37	36.3	33/37	89.2	
	Autumn	26	25.5	26/26	100.0	
	Winter	30	29.4	28/30	93.3	
	Spring	9	8.8	8/9	88.9	
Breed group (*n* = 97)						0.8429
	Herding	8	8.3	8/8	100.0	
	Hound	14	14.4	13/14	92.9	
	Non-sporting	7	7.2	6/7	85.7	
	Sporting	16	16.5	14/16	87.5	
	Terrier	13	13.4	13/13	100.0	
	Toy	21	21.7	19/21	90.5	
	Working	18	18.6	17/18	94.4	
Age group (*n* = 98)						0.3303
	<2 years	17	17.4	16/17	94.1	
	2–4 years	27	27.6	24/27	88.9	
	>4–7 years	28	28.6	25/28	89.3	
	>7–10 years	26	26.5	26/26	100.0	
Sex (*n* = 98)						0.7053
	Female	62	63.3	58/62	93.6	
	Male	36	36.7	33/36	91.7	
Organ system (*n* = 86)						0.886
	Ear	19	22.1	16/19	84.2	
	GIT	13	15.1	12/13	92.3	
	Uro-genital	33	38.4	33/33	100.0	
	Respiratory	8	9.3	7/8	87.5	
	Skin	13	15.1	12/13	92.3	

*x, Number of multidrug resistant isolates; n, Number of samples tested; MDR, Multidrug resistant isolates*.

* *p-values are Fisher's exact p-values except for Year which is Cochran-Armitage Exact Trend test*.

#### Rationale of Data Analysis and Data Analysis

##### Rationale of data analysis

Since the outcome (XDR status; Yes = 1/No = 0) was a binary variable, a binary logistic regression model was fitted to the data to investigate the association between the explanatory variables and the outcome (XDR).

### Data Analysis

All the statistical analyses were performed in SAS 9.4 statistical package (SAS Institute Inc., Cary, NC, USA).

#### Descriptive Statistics

The age of the dogs from which samples were obtained was not normally distributed (Shapiro-Wilk W = 0.958; *p* = 0.0035), and therefore the median and interquartile range (IQR) were calculated. Percentages or proportions of categorical variables were calculated and presented as tables.

#### Inferential Statistics

##### Models to identify predictors of XDR

Since all isolates in the study dataset were resistant to at least one antimicrobial (AMR) and almost all isolates were multidrug resistant (MDR), the associations between predictor variables; year, season, breed group, age group, sex, and organ system and AMR or MDR were not assessed. Therefore, only the association between the predictor variables listed above and one outcome variable; XDR status (Yes = 1/No = 0) was assessed using a logistic regression model.

##### The model building

Building of the models was done in two steps: the 1st step involved building univariable logistic regression models to identify potential predictors associated with the outcome at a relaxed α ≤ 0.20. Variables with *p* ≤ 0.20 in the univariable model were selected for inclusion in the multi-variable model. The 2nd step involved fitting a multivariable logistic regression model using manual backwards selection method with the level of significance set at α ≤ 0.05.

Confounding was assessed by comparing the change in model coefficients with and without the suspected confounders. If the removal of a suspected confounding variable resulted in a ≥20% change in the coefficient of any variable in the model, the variable that had been removed was considered a confounder and was thus retained in the model regardless of whether it was significantly associated with the outcome variable or not.

Odds ratios (ORs) and their 95% confidence intervals were computed for variables included in the final model. Hosmer-Lemeshow goodness-of-fit test was used to assess model fit.

## Results

### The Number of Enterococcus Isolates

The proportions of *Enterococcus* species isolated from dog clinical samples submitted to the bacteriology laboratory between 2007 and 2011 are presented in Table 1. A total of 102 *Enterococcus* spp. were isolated over the study period. The highest percentage were isolated in 2009 (48%) and the lowest numbers were isolated in both 2007 and 2011.

Stratified by season of isolation, most of the 102 *Enterococcus* isolates, were isolated in summer (36.3%). In terms of breeds, 21.7% of the isolates came from toy breeds, while the working breeds contributed the least number of isolates.

Dogs aged 5–7 and 2–4 years old yielded the highest number of isolates (28.6 and 27.6%, respectively). The lowest number of isolates were from dogs aged <2 years.

Stratified by sex, majority of the 102 isolates, were recovered from female dogs (63.3%). When the study population was stratified by sample type, the uro-genital specimens contributed 38.4% of isolates, which was the highest, followed by ear specimens that contributed 22.1% of the isolates. The respiratory system contributed the least number of isolates.

### Multi-Drug Resistant Isolates

More than 80% of the *Enterococcus* isolates included in this study were MDR. As shown in Table 1, the number of isolates that were MDR did not vary significantly across years or seasons. Likewise, there was no significant variations in MDR across breed groups, age groups, sex, and specimen types.

### Antimicrobial Drug Resistance (AMR)

All the *Enterococcus* isolates included in the study were resistant to at least one antimicrobial agent, and a very high proportion of these isolates exhibited resistance against the following antimicrobial groups: Lincosamide (94.1%), aminoglycosides (92.2%), fluoroquinolones (85.3%), and β-lactams (78.4) (Table 2).

**Table 2:** Antimicrobial resistance profile of *Enterococcus* isolates from dogs tested at a teaching veterinary hospital in South Africa, 2007–2012.

**Antimicrobial Group/Class**	**Category of antimicrobial**	**Antimicrobial**	**x/n**	**Percentage**
**β-lactam**			**80/102**	**78.4**
		Carbenicillin	2/3	66.7
		Penicillin-G	45/99	45.5
		Ampicillin	42/102	41.2
	Carbapenem	Imipenem	2/2	100.0
	Cephalosporin	Ceftazidime	2/2	100.0
		Cephalothin	68/102	66.7
	Amoxicillin-clavulanic acid	Amoxicillin-clavulanic acid	21/93	22.6
**Aminoglycoside**			**94/102**	**92.2**
		Amikacin	89/102	87.3
		Gentamicin	72/101	71.3
		Kanamycin	86/99	86.9
		Tobramycin	2/2	100.0
**Amphenicols**	**Chloramphenicol**	**Chloramphenicol**	**21/80**	**26.3**
**Fluoroquinolone**			**87/102**	**85.3**
		Orbifloxacin	86/101	85.2
		Enrofloxacin	58/100	58.0
**Lincosamide**			**96/102**	**94.1**
	Lincosamide	Lincomycin	94/101	93.1
	Aminogycoside-Lincosamide	Aminogycoside-Lincosamide	78/102	76.5
**Macrolide**	**Macrolide**	**Tylosin**	**42/100**	**42.0**
**Potentiated sulfonamide**	**Potentiated sulfonamide**	**Trimethoprim-sulfamethoxazole**	**29/101**	**28.7**
**Tetracycline**	**Tetracycline**	**Oxytetracycline**	**58/102**	**56.9**

*x, Number of antimicrobial resistant isolates; n, Number of isolates tested*.

Over half of the isolates were resistant to each of the following individual drugs: carbenicillin (66.7%), cephalothin (66.7%), amikacin (87.3%), gentamicin (71.3%), kanamycin (96.9%), tobramycin (100%), orbifloxacin (85.2%), enrofloxacin (58.0%), lincomycin (93.1%), aminogycoside-lincosamide (76.5%), and doxycycline (56.9%) (Table 2). On the contrary, relatively low proportions of isolates exhibited resistance against amphenicols (26.3%), Amoxicillin-clavulanic acid (22.6%) and potentiated sulfonamides (28.7%) (Table 2).

Results in Table 3 show that very high levels of resistance were consistently observed against the following drugs for the duration of the study: amikacin, cephalothin, kanamycin, lincomycin, aminoglycoside-lincosamide, and orbifloxacin. However, the number of isolates that were resistant to other antimicrobials fluctuated over the study period. Additionally, the proportion of isolates resistant to antimicrobials such as amikacin, ampicillin, and penicillin G, seemed to increase until 2010 and then dropped either drastically or slightly in 2011.

**Table 3:** Distribution of AMR *Enterococcus* isolates from dogs presented at a teaching veterinary hospital by year for the period 2007-2011.

	**Year**					
**Drug^†^**	**2007** **%^a^ (x/n)^b^**	**2008** **%^a^ (x/n)^b^**	**2009** **%^a^ (x/n)^b^**	**2010** **%^a^ (x/n)^b^**	**2011** **%^a^ (x/n)^b^**	**Total** **%^a^ (x/n)^b^**
Amikacin	81.8 (9/11)	88.2 (15/17)	85.7 (42/49)	100 (14/14)	81.2 (9/11)	87.3 (89/102)
Ampicillin	18.2 (2/11)	29.4 (5/17)	42.9 (21/49)	71.4 (10/14)	36.4 (4/11)	41.2 (42/102)
Cephalothin	72.7 (8/11)	52.9 (9/17)	67.4 (33/49)	78.6 (11/14)	63.6 (7/11)	66.7 (68/102)
Chloramphenicol	100 (1/1)	41.7 (5/12)	13.0 (6/46)	57.1 (8/14)	14.3 (1/7)	26.3 (21/80)
Oxytetracycline	81.8 (9/11)	47.1 (8/17)	51.0 (25/49)	78.6 (11/14)	45.6 (5/11)	56.9 (58/102)
Enrofloxacin	70 (7/10)	41.2 (7/17)	54.2 (26/48)	78.6 (11/14)	63.6 (7/11)	58.0 (58/100)
Gentamicin	63.6 (7/11)	52.9 (9/17)	71.4 (35/49)	100 (13/13)	72.7 (8/11)	71.3 (72/101)
Kanamycin	90.9 (10/11)	81.3 (13/16)	81.3 (39/48)	100 (13/13)	100 (11/11)	86.9 (86/99)
Lincomycin	90 (9/10)	94.1 (16/17)	91.8 (45/49)	100 (14/14)	90.9 (10/11)	93.1 (94/101)
Aminogycoside-Lincosamide	100 (11/11)	52.9 (9/17)	71.4 (35/49)	92.9 (13/14)	90.9 (10/11)	76.5 (78/102)
Orbifloxacin	81.8 (9/11)	70.6 (12/17)	87.5 (42/48)	92.9 (13/14)	90.9 (10/11)	85.1 (86/101)
Penicillin G	37.5 (3/8)	35.3 (6/17)	44.9 (22/49)	71.4 (10/14)	36.4 (4/11)	45.5 (45/99)
Trimethoprim-sulfamethoxazole	27.3 (3/11)	29.4 (5/17)	18.8 (9/48)	71.4 (10/14)	18.2 (2/11)	28.7 (29/101)
Amoxicillin-clavulanic acid	22.2 (2/9)	12.5 (2/16)	9.3 (4/43)	57.1 (8/14)	45.5 (5/11)	22.6 (21/93)
Tylosine	40 (4/10)	35.3 (6/17)	38.8 (19/49)	61.5 (8/13)	45.5 (5/11)	42.0 (42/100)

a Percentage of resistant isolates;

b *x, number of resistant isolates; n, number of isolates assessed*.

† *Imipenem, ceftazidime, and tobramycin are not included here due to low number of samples*.

### Pan-Drug Resistance (PDR)

Eight percent (7.84%; *n* = 8/102) of the isolates in this study exhibited PDR, and were isolated from uro-genital (*n* = 4), ear (*n* = 2), and GIT (*n* = 1) samples. But as shown in Table 4, two (*n* = 2) of the eight (*n* = 8) PDR isolates had missing organ information.

**Table 4:** The year of isolation and characteristics of dogs with PDR *Enterococcus* species infections whose samples were processed at the veterinary academic hospital, 2007-2012.

**Year**	**Breed**	**Age (Months)**	**Sex**	**Organ**
2008	Minature Doberman Pinscher	36	M	Uro-genital
2008	Maltese	5	M	–
2009	Poodle	11	F	–
2010	Dachshund	72	F	Uro-genital
2010	Husky	2	F	Uro-genital
2010	Chihuahua	108	M	GIT
2010	Spaniel	106	F	Ear
2011	Labrador retriever	14	F	Ear

As was the case with XDR isolates, most the PDR isolates (62.5%; *n* = 5/8) came from female dogs, and from the uro-genital tract (37.5%; *n* = 3/8). The median age of dogs with PDR infections was 36 months (3 years). However, overall, the range of the age of dogs included in this study was 2 to 108 months (Table 4).

### Predictors of Extensive-Drug Resistance (XDR)

Overall, 35.3% (*n* = 36/102) of the *Enterococcus* isolates tested, exhibited XDR. Only year had a statistically significant association with XDR (*p* = 0.0019) and hence it was the only variable included in the final XDR model. The odds of isolates being XDR were lower for isolates obtained in 2008 (OR: 0.109, *p* = 0.012), 2009 (OR: 0.101, *p* = 0.002), and 2010 (OR: 0.078, *p* = 0.014) compared to those obtained 2011 (Table 5).

**Table 5:** Distribution of XDR enterococci from dog specimens submitted by the teaching hospital in South Africa between 2007 and 2011, and the association between predictor variables and the outcome (XDR).

	**Univariable model**				**The final model**			
**Variable**	**x/n**	**%**	**95% CI**	*p*-value^*^	**OR**	**95% CI**	***p*-value**	***p*-value^d^**
Year (*n* = 102)				0.0019			0.019	0.999
2007	6/11	54.6	23.4, 83.3		0.273	0.046, 1.616	0.152	
2008	4/17	23.5	6.8, 49.9		0.109	0.019, 0.612	0.012	
2009	13/49	26.5	14.9, 41.1		0.101	0.024, 0.422	0.002	
2010	11/14	78.6	49.2, 95.3		0.078	0.010, 0.590	0.014	
2011	2/11	18.2	2.3, 51.8		Re			
Season (*n* = 102)				0.8454				
Summer	14/37	37.8	22.5, 55.2					
Autumn	10/26	38.5	20.2, 59.4					
Winter	10/30	33.3	17.3, 52.8					
Spring	2/9	22.2	2.8, 60.0					
Breed group (*n* = 97)				0.7714				
Herding	3/8	37.5	8.5, 75.5					
Hound	5/14	35.7	12.8, 64.9					
Non-sporting^†^	4/7	57.1	18.4, 90.1					
Sporting	7/16	43.8	19.8, 70.1					
Terrier	5/13	38.5	13.9, 68.4					
Toy	8/21	38.1	18.1, 61.6					
Working	4/18	22.2	6.4, 47.6					
Age group (*n* = 98)				0.1559				
<2 years	9/17	52.9	27.8, 77.0					
2–4 years	10/27	37.0	19.4, 57.6					
5–7 years	6/28	21.4	8.3, 41.0					
>8 years	11/26	42.3	23.4, 63.1					
Sex (*n* = 98)				0.0485				
Female	27/62	43.5	31.0, 56.7					
Male	8/36	22.2	10.0,39.2					
Organ system (*n* = 86)				0.3137				
Ear	7/19	36.8	16.3, 61.6					
GIT	5/13	38.5	13.9, 68.4					
Uro-genital	16/33	48.5	30.8, 66.5					
Respiratory	1/8	12.5	0.3, 52.7					
Skin	3/13	23.1	5.0, 53.8					

*x, Number of extensively resistant isolates; n, Number of samples tested*.

* *p-values are Fisher's exact p-values except for Year which is Cochran-Armitage Exact Trend test*.

† *Non-sporting dogs come from a wide variety of backgrounds and include: American Eskimo Dog, Bichon Frise, Boston Terrier, Bulldog, Chinese Shar-Pei, Chow Chow, Coton De Tulear, Dalmatian, Finish Spitz, French Bulldog, Keeshond, Lhasa Apso, Lowchen, Norwegian Lundhund, Poodle, Schipperke, Shiba Inu, Tibetan Spaniel, Tibetan Terrier, and Xoloitzcuintli*.

d *Hosmer-Lemeshow Goodness of fittest p-value*.

## Discussion

In this study, we investigated using data from samples submitted to the bacteriology laboratory for isolation of enterococci and assessing antimicrobial susceptibility of enterococci isolates. This investigation revealed that most of the enterococci isolates were from urino-genital samples. We also observed that enterococci were isolated from various organ systems. The study also demonstrated that a high number of isolates were resistant to at least one antimicrobial and that a high number of isolates were also MDR and XDR. However, pan-drug resistance was not as widespread. Year was the only explanatory variable that was associated with XDR. Of noteworthy, is that results reported here showed that resistance to antimicrobials recommended for the treatment of enterococci such as amoxicillin-clavulinic Acid combination (23%), ampicillin (41%), and penicillin-G (46%), exhibited relatively lower levels of resistance compared to other antimicrobials tested in this study.

Dogs are commonly colonized by antimicrobial drug resistant enterococci and thus act as a reservoir for resistant enterococci for humans. Therefore, continued and increasing use of antimicrobials in dogs increases the risk of commensal bacteria such as enterococci developing resistance and transferring the resistance to humans. Yet emphasis has focused on food animals as source of resistance transfer to humans, with little attention given to the role of pets or companion animals as a possible source of resistant organisms and/or genes that mediate resistance (25, 50, 51).

### Enterococci Isolates

Out of a total of 102 enterococci isolated from the clinical samples from dogs, the largest proportion (38.4%) were from urogenital samples. According to Weese et al. (52) and Kajihara et al. (53) enterococci are common causative pathogens in complicated urinary tract infections. Since the samples are from dogs attending a referral hospital it is possible that many of these cases had been referred to the hospital with urinary tract infection. Unfortunately, the authors were not able to verify this due to lack of data documenting why the dogs were presented for veterinary care at the teaching hospital in question.

However, Jackson et al. (50) have noted that certain enteroccocal species tend to be predominantly isolated from the rectum as opposed to other organ systems. There are several explanations for the observed difference between our findings and those reported by Jackson et al. (50). The first being that, the present study was not designed to establish the prevalence of enterococci by origin, and secondly unlike the study by Jackson et al. (50), the present study considered enterococci as a genus and did not focus on specific species such as *E. faecalis* and *E. faecium*. Thirdly, it is known that both *E. faecalis* and *E. faecium* have a primary intestinal reservoir in vertebrate animals. The other possible explanations for the difference between our findings and those of Jackson et al. (50), is that the later did not collect samples from the urogenital organs, and furthermore, while we used clinical samples, the study by Jackson and colleagues used samples from healthy dogs.

Isolation of enterococci from several organ systems, is consistent with the observation that enterococci do not only inhabit several habitats, but also inhabit various parts of the body of dogs (50). Therefore, results reported here confirm that enterococci can infect several animal organ systems and cause several infections such as urinary tract infections, hepatobiliary sepsis, endocarditis, surgical wound infections, bacteraemia, and neonatal sepsis (25, 53, 54).

### Antimicrobial Drug Resistance Among Enterococci Isolates

In the study by Ossiprandi and Zerbini (51), a very high level of resistance was observed in various *Enterococci* spp. against antimicrobials such as erythromycin, enrofloxacin, and tetracycline. Similarly in a study conducted in Japan (25), the authors also observed that the most active resistance among enterococci from dogs was against erythromycin (44.2%) and tetracycline (44.2%), while considerable resistance was observed against lincomycin (41.6%), kanamycin (32.2%), and gentamycin (32.2%). This is consistent with our findings that show that all isolates were resistant to at least one antimicrobial agent, with very high prevalence observed against antimicrobials such as lincomycin (93.1%), aminoglycosides-lincosamide (92.2%), oxytetracycline (56.9%), orbifloxacin (85.2%), and carbenicillin (66.7%). According to Kataoka et al. (25) such high prevalence of resistance should be expected because dogs treated with antimicrobials are commonly colonized with antimicrobial resistant enterococci.

Moreover, since the isolates used in this study were from a teaching hospital which, also doubles as a referral hospital, it is possible that most of the cases from which the samples were drawn, had already been exposed to antimicrobial treatment by the time they were presented at the hospital. Furthermore, such a high occurrence of resistance among enterococci should be expected given that enterococci relative to streptococci are known to be intrinsically resistant to most commonly used antimicrobials agents and that enterococci have genetic capability of acquiring, conserving and disseminating genetic traits including genetic resistance determinants among themselves (16, 55). According to Watson (54) there has been an increase in drug-resistant *E. faecalis* strains, and that many antibiotics are no longer effective against infections caused by these bacteria.

The high prevalence of resistance (94.1%) observed against lincosamide, was expected. This is because enterococci are known to be intrinsically resistant to drugs such as clindamycin that inhibit protein synthesis at the chain elongation step by interfering with the transpeptidation of the 50S ribosomal subunit. Enterococci are also able to exhibit native resistance to clinically achievable concentrations of aminoglycosides, a feature which precludes their use as single agents. Therefore, the very high levels of resistance against aminoglycoside (92.2%) observed in this study suggest that aminoglycosides may be of very limited therapeutic importance in clinical practice at the hospital under study (16).

Worth noting, was the observation of resistance albeit lower levels (in comparison to other drugs presented in this study) of resistance against drugs such as amoxicillin/clavulanic acid combination, ampicillin, tylosin, and penicillin G, that ordinarily constitute the empirical and definitive antimicrobial treatment for enterococci (16, 55). This relatively low resistance level against these antimicrobials is nonetheless to be expected because all enterococci organisms tend to display low-affinity penicillin-binding proteins, which leads to decreased susceptibility and hence the observed low levels of resistance against these drugs (16).

Resistance levels of 41% against ampicillin is concerning given that it is the drug of choice for empirical treatment of enteroccocal infections. According to Damborg et al. (23) ampicillin resistance is a marker for hospital—associated *E. faecium*. Considering this, it is possible that the isolates under study were predominantly *E. faecium*. The fact that the isolates were from cases treated at a referral hospital further gives credence to the suggestion that the isolates under study were predominantly *E. faecium*. The suggestion that most isolates recovered in this study could have been *E. faecium*, is also supported by Kristich et al. (16), who are of the view that ampicillin resistance tends to be rare in *E. faecalis*, but widespread among *E. faecium*. However, this could not be confirmed since the laboratory did not carry out speciation of the isolates.

Despite the observed 41% resistance against ampicillin, it cannot be precluded from the treatment of enteroccocal infections among patients presented at the teaching hospital under study (16). Which means that ampicillin can thus still be considered the drug of choice for the treatment of enterococci. However, this is only true if the organisms do not have other mechanisms for high level resistance.

Enterococci are known to be tolerant to the bactericidal activity of cell-wall active agents such as β-lactams, meaning that while enterococci can be inhibited by clinically achievable concentrations of the antibiotic, only concentrations far in excess of the inhibitory concentrations are able to kill the bacteria. For this reason, β-lactams are normally used in combination with aminoglycosides to enhance the synergistic bactericidal activity of—β-lactams (16, 55). Due to the inaccessibility of bacteria to the mammalian immune system because of its location within the cardiac vegetations, the synergism between β-lactams and aminoglycosides is important in the treatment of several conditions including endocarditis that require enhanced synergistic bactericidal activity (16). However, the high levels of resistance observed against aminoglycosides in this study, undermines the practice of taking advantage of the synergism between cell-wall active agents and aminoglycosides in the treatment of serious enterococci infections caused by enterococci.

According to Kristichet al. (16) and the CLSI (56), while trimethoprim-sulfamethoxazole, cephalosporins, aminoglycosides, and clindamycin, may appear to be active *in vitro* against enterococci, they have not proved to be successful in animal models. In view of this, even though only 28.7% of the isolates in this study were resistant to sulfamethoxazole-trimethoprim, this should not be interpreted to mean that this drug combination could be effective clinically. In fact, the CLSI recommends that enterococci isolates should not be reported as being susceptible even if found to have been sensitive to sulfamethoxazole-trimethoprim (46).

While high levels of resistance were consistently observed against drugs such as amikacin, cephalothin, kanamycin, lincomycin, aminoglycoside-lincosamide, and orbifloxacin for the duration of the study, the number of isolates that were resistant to other antimicrobials tended to fluctuate. This could be a reflection of antimicrobial prescription practices at the hospital. The authors postulate that clinicians could be using antimicrobials for a certain period, leading to a build up of resistance, and then withdraw the antimicrobial in question when resistance against the antimicrobial is picked frequently. During this time, the antimicrobial, which has been withdrawn, is replaced with antimicrobials from other classes with different activity spectrum or mechanisms of action. However, due to lack of data on treatment practices and patterns at the hospital, it is not possible to confirm this hypothesis.

### Multidrug, Extensive, and Pan-Drug Resistance

The level of XDR (35.5%) and MDR (93.1%) observe in this study, could be explained by the fact that enterococci in addition to being intrinsically resistant and tolerant, are ordinarily capable of rapidly acquiring resistance to virtually any antimicrobial agent put into clinical use. For example, following the introduction of chloramphenicol, erythromycin, and tetracyclines, resistance to these drugs quickly emerged to the extent that prevalences of resistance attained against these drugs precluded their empirical use (16).

All XDR isolates exhibited resistance against ampicillin or other related drugs such as amoxicillin, drugs of choice in the treatment of these infections. This was expected given that β-lactams are the usually the first line of defense in the treatment against enterococci. This being a referral hospital, it is highly likely that all the dogs included in this study had previously been treated with (unknown) antibiotics.

### Factors That Predicted XDR Among Enterococci Isolates

Year was the only explanatory factor that was associated with XDR. Except for isolates obtained in 2007, isolates from other earlier years (2008-2010), had significantly lower odds of being resistant compare to isolates obtained in 2011. Which suggests that the level of resistance among enterococci isolates was more likely to be higher in 2011 as compared to earlier years. A similar trend was observed among *Staphylococcus aureus* isolates in a study by the same authors in which increasing resistance to certain antimicrobials over the same period (2007-2011) was observed. Furthermore, a similar trend (increase in the number of isolates that were resistant to certain antimicrobials) was observed in *Staphylococcus pseudintermedius* over the same period (57). The reason for the observed lower odds of resistance in earlier years compared to 2011 are not clear. However, the authors are of the view that this could reflect the changing patterns in the use of antimicrobials at the hospital with a higher selection pressure exerted in 2011 compared to previous years. In view of this, data is needed to investigate prescription practices and patterns at the teaching hospital in question, so that measures can be implemented to reverse the trend and safeguard the efficacy of antimicrobials.

### Limitations of the Study

This being a retrospective study that used secondary data, only variables available in the database could be investigated. This therefore, limited the variables that could be investigated in the logistic models. Furthermore, since there was no species differentiation, it was not possible to investigate differences in resistance between the two main *Enterococcus* species commonly isolated from dogs; *E. faecalis* and *faecium*. Moreover, the laboratory does not routinely test for vancomycin and therefore resistance status of this glycopeptide (useful for treatment of drug resistant human enteroccocal infections) could not be assessed. The laboratory uses a standard panel of antimicrobials for all susceptibility tests and hence the panel included a couple of drugs against which enterococci exhibit intrinsic resistance, which has the potential to skew the results. Lastly, the relatively low sample size implies that study findings should be interpreted with caution. These limitations notwithstanding, the findings of this study are useful in resource poor settings where it is not possible to implement regular AST to guide selection of antimicrobials for use where first line antimicrobials have failed.

## Conclusion

The high levels of AMR, MDR, and XDR observed in this study are of serious public health concern from a one-health perspective. Therefore, efforts to decrease the prevalence of resistance among these organisms with zoonotic potential and the ability to spread elements mediating resistance to other organisms are urgently needed. The identified resistance levels against the different antimicrobial groups may be useful in guiding clinicians in making treatment decisions especially in resource scarce settings where it is not always possible to base treatment on AST.

## Data Availability Statement

The datasets presented in this article are not readily available because they belong to a third party (the Bacteriology Laboratory of the Veterinary Academic Hospital, University of Pretoria). The authors do not have legal rights to share the data. Requests to access the datasets should be directed to Prof. Vinny Naidoo at vinny.naidoo@up.ac.za.

## Author Contributions

JO was involved in study design, data analysis, interpretation of results, writing of manuscript as well as extensive editing of the manuscript. DQ was involved in study design and data management and statistical analyses and interpretation as well as reviewing of the manuscript draft. AO was involved in study design, data analysis and interpretation as well as editing of the manuscript. All authors read and approved the final manuscript.

## Conflict of Interest

The authors declare that the research was conducted in the absence of any commercial or financial relationships that could be construed as a potential conflict of interest.

## Abbreviations

AKC: American Kennel Club
AMR: Antimicrobial resistance
CADFP: Carnegie African Diaspora Fellowship Program
CI: Confidence Interval
CLSI: Clinical and Laboratory Standards Institute
MDR: Multidrug resistance
PDR: Pan-drug resistance
UK: United Kingdom
US: United States
USA: United States of America
XDR: Extensive drug resistance.
